# Understanding Starch Metabolism in Pea Seeds towards Tailoring Functionality for Value-Added Utilization

**DOI:** 10.3390/ijms22168972

**Published:** 2021-08-20

**Authors:** Bianyun Yu, Daoquan Xiang, Humaira Mahfuz, Nii Patterson, Dengjin Bing

**Affiliations:** 1 Aquatic and Crop Resource Development Research Centre, National Research Council Canada, 110 Gymnasium Place, Saskatoon, SK S7N 0W9, Canada; Daoquan.Xiang@nrc-cnrc.gc.ca (D.X.); Hmahf026@uOttawa.ca (H.M.); Nii.Patterson@nrc-cnrc.gc.ca (N.P.); 2 Department of Biology, Faculty of Science, University of Ottawa, 30 Marie Curie, Ottawa, ON K1N 6N5, Canada; 3 Lacombe Research and Development Centre, Agriculture and Agri-Food Canada, 6000 C and E Trail, Lacombe, AB T4L 1W1, Canada; dengjin.bing@agr.gc.ca

**Keywords:** starch metabolism, genetics, genomics, functionality, applications, pea (*Pisum sativum* L.)

## Abstract

Starch is the most abundant storage carbohydrate and a major component in pea seeds, accounting for about 50% of dry seed weight. As a by-product of pea protein processing, current uses for pea starch are limited to low-value, commodity markets. The globally growing demand for pea protein poses a great challenge for the pea fractionation industry to develop new markets for starch valorization. However, there exist gaps in our understanding of the genetic mechanism underlying starch metabolism, and its relationship with physicochemical and functional properties, which is a prerequisite for targeted tailoring functionality and innovative applications of starch. This review outlines the understanding of starch metabolism with a particular focus on peas and highlights the knowledge of pea starch granule structure and its relationship with functional properties, and industrial applications. Using the currently available pea genetics and genomics knowledge and breakthroughs in omics technologies, we discuss the perspectives and possible avenues to advance our understanding of starch metabolism in peas at an unprecedented level, to ultimately enable the molecular design of multi-functional native pea starch and to create value-added utilization.

## 1. Introduction

Starch or amylum is a polysaccharide synthesized in plants; it remains at the very epicenter of the food and feed chains, as well as a renewable source of industrial raw materials [1]. Starch is composed of two distinct types of glucose polymers: the linear and lightly branched amylose comprising glucose moieties linked together by α-1,4-glycosidic bonds, and the highly branched amylopectin with shorter α-1,4-linked glucan chains connected by α-1,6-glycosidic bonds [2]. In higher plants, starch is synthesized through polymerization of glucose in plastids, including transient starch produced in chloroplasts in photosynthetic tissues and long-term storage starch synthesized in amyloplasts in non-photosynthetic tissues such as seeds, storage roots and tubers [3]. Transient starch synthesized from photosynthates during the day is degraded at night to provide energy and sustain metabolism. Storage starch is kept for long periods of time and ready for remobilization during germination, sprouting or regrowth [1].

Field pea (*Pisum sativum* L.) belongs to the legume family and is a major pulse crop with a production yield of 14.2 million tons globally in 2019 (FAO 2019, http://www.fao.org (accessed on 31 July 2021)). Known as a healthy pulse crop, peas have historically been part of the human diet as they are an excellent source of protein, starch, fiber, antioxidants and micronutrients. More recently, peas have gained considerable attention due to their potential health-promoting benefits in reducing the risk of chronic health conditions [4]. In addition, as the food industry is seeking diversified sources of plant-based proteins, demands for pea protein are growing and expanding into new end-use applications, for a healthier alternative to more traditional food ingredients. Starch accounts for about 50% of pea seed dry weight [4] and is a by-product of protein processing. The global pea protein market was valued at USD 32.09 million in 2017, and is projected to reach USD 176.03 million by 2025 (https://www.alliedmarketresearch.com/pea-protein-market (accessed on 31 July 2021)). Consequently, the volume of pea starch will dramatically grow at a rate double that of pea protein. The explosion in demand for pea protein has led to a surplus of pea starch, which poses significant challenges to the pea processing industry. This highlights the importance of increasing the value-added utilization of pea starch. The successful utilization of starch in various applications depends largely on its physicochemical and functional properties such as gelatinization/pasting, viscosity and swelling power [5]. These properties are determined by the content of amylose and amylopectin, amylopectin chain length and granule structure. 

Starch biosynthesis is a complicated process involving the coordinated interactions of a suite of key biosynthetic enzymes with multiple isoforms [2,6]. The complexity of the starch metabolic process is augmented by recently identified non-enzymatic proteins that play regulatory roles through interacting with biosynthetic enzymes, their substrates and/or other cellular structures [7]. The development of peas with altered or modified starch metabolism could be beneficial to change the starch composition and content; to prevent or increase starch degradation; or to modify starch structure to enhance or diversify its functionality in food applications and as an industrial material. The understanding of the starch biosynthetic pathway in peas has been advanced by the characterization of the mutants through individual steps of the pathway, as well as the identification and isolation of genes encoding the key enzymes in the pathway. Limited modifications of pea starch have been achieved by genetically modulating the previously identified genes. This reveals the current gaps in the understanding of the genetic mechanisms underlying starch metabolism, and the relationship of starch structure and functional properties. The identification and characterization of novel genes and novel allelic variations in known genes may provide further gene targets for custom modulating starch composition, structure and, thus, functionality. The availability of pea starch with diverse functional properties suitable to different specific industrial applications will promote the use of native pea starch, leading to simpler and more eco-friendly processing steps, as well as greater cost benefits for pea processors.

## 2. Starch Metabolic Pathway

As the principal storage carbohydrate, starch plays important roles during the life cycle of a plant. The metabolism of starch begins with the unloading of sucrose to sink tissues from the phloem by sucrose transporters. Sucrose is then cleaved by sucrose synthase (SuSy) into uridine diphosphate glucose (UDP-Glc) and fructose to provide a carbon skeleton for the synthesis of adenosine 5′-diphosphate-glucose (ADP-Glc), the soluble precursor for starch biosynthesis [8]. The characterization of naturally occurring or induced mutants with altered starch content, composition and/or properties is an instrumental strategy used to identify the genes encoding starch biosynthetic enzymes in peas. These studies also demonstrated that the different isoforms of these enzymes likely play various specific roles in determining the complex structure and properties of pea starch. Starch degradation, however, has been extensively reviewed elsewhere [8,9] and is not a focus of this article.

### 2.1. Core Starch Biosynthetic Enzymes

**Figure 1:**
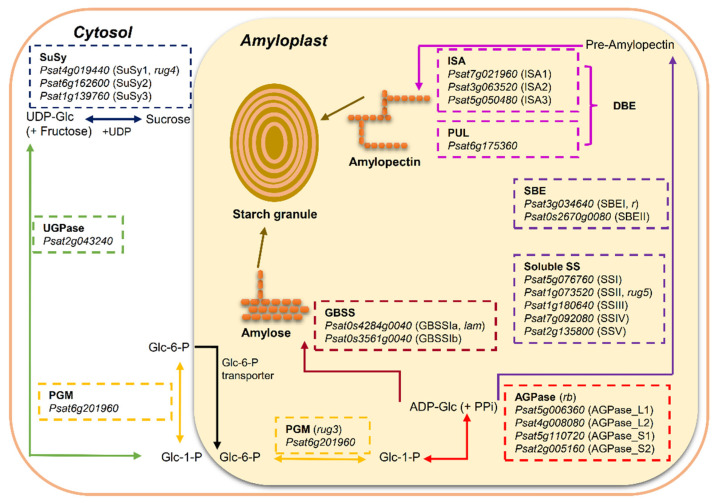
Overview of starch biosynthesis in the heterotrophic tissue (amyloplast) of pea (*Pisum sativum* L.) seeds based on the literature [2,8,10]. Sucrose is metabolized to hexose-phosphates via reversible conversion into fructose and uridine diphosphate glucose (UDP-Glc) by sucrose synthase (SuSy) to provide carbon skeleton. UDP-Glc is metabolized to glucose 1-phosphate (Glc-1-P) by UDP-Glc pyrophosphorylase (UGPase) and then reversibly converted to glucose 6-phosphate (Glc-6-P) by phosphoglucomutase (PGM). Glc-6-P is the only substrate imported into the amyloplast for starch synthesis in pea seeds. In the amyloplast, Glc-1-P, converted from Glc-6-P by plastidial PGM, serves as the precursor for the synthesis of adenosine 5′-diphosphate-glucose (ADP-Glc)—the soluble precursor for starch biosynthesis by ADP-Glc pyrophosphorylase (AGPase). Starch is composed of two distinct types of glucose polymers: the linear and lightly branched amylose and the highly branched amylopectin. Granule bound starch synthase (GBSS) is primarily responsible for the synthesis of amylose and extra-long chains of amylopectin. Amylopectin is synthesized through the coordinated actions of a suit of enzymes: soluble starch synthase (SS), starch branching enzyme (SBE) and starch debranching enzyme (DBE). Putative candidate gene for each enzyme isoform was retrieved from reference genome assembly of pea cultivar Caméor (https://urgi.versailles.inra.fr/Species/Pisum/Pea-Genome-project (accessed on 31 July 2021)).

Plants produce starch by converting glucose 1-phosphate (Glc-1-P) to ADP-Glc and then liberating the glucosyl unit to a growing glucan chain. These processes involve the coordinated actions of four major classes of enzymes: ADP-Glc pyrophosphorylase (AGPase), starch synthases (SS), including soluble starch synthases (SS) and granule-bound starch synthases (GBSS); starch branching enzymes (SBE); and starch de-branching enzymes (DBE) (Figure 1).

In starch-storing organs, such as the endosperms of cereal grains and the embryos of legume seeds, sucrose derived from photosynthesis in the leaves is remobilized to provide a substrate for starch synthesis [11]. In pea seeds, sucrose is converted into glucose 6-phosphate (Glc-6-P) in the cytosol, and then Glc-6-P enters the amyloplast via a specific transporter in the membrane. In the amyloplast, the conversion of Glc-6-P to glucose 1-phosphate (Glc-1-P) is subsequently followed by the formation of ADP-Glc via the enzyme AGPase (Figure 1). ADP-Glc is the substrate and glucosyl donor for the starch synthases that synthesize the starch polymers. It has been demonstrated that Glc-6-P is the only substrate imported into amyloplast for starch synthesis in pea seeds [10]. This is different from the pathway of ADP-Glc synthesis in the endosperms of cereal grains where most (65–95%) of the ADP-Glc is made in the cytosol by a distinct cytosolic form of AGPase and then transported into the amyloplast [2,12]. Alternatively, a minor pathway in cereal grains leads to the production of ADP-Glc by plastidial AGPase, with Glc-1-P as the imported substrate into the amyloplast [10]. 

The generation of ADP-Glc is the first committed step which is catalyzed by AGPase, a heterotetrameric enzyme consisting of two large subunits encoded by small multiple gene families and two small catalytic subunits encoded by one or two genes [13]. AGPase is allosterically regulated by both inorganic phosphate (Pi, an inhibitor) and 3-phosphoglyceric acid (3-PGA) (an activator). However, the relative sensitivity of AGPases to these allosteric effectors varies depending on the tissue and plastid type [2]. Evidence from endosperms of wheat [2] and barley [14] suggests that AGPase is relatively insensitive to Pi-inhibition and 3-PGA activation. In the legume species *Vicia faba* L., the AGPase activity in developing cotyledons is insensitive to 3-PGA, while the activity from leaves is. However, the AGPase activities from both tissues are sensitive to Pi-inhibition [15]. The small subunits alone can perform catalytic activity, while the larger units have no catalytic activity but can modulate the sensitivity of the small ones to the allosteric regulation [10]. Variation in the subunit composition, and consequently variation in the kinetic properties of AGPase, leads to a difference in regulation of flux through the pathways in different organs. A high level of sequence conservation exists in small subunits among different plant species, while more divergence is shown in the large subunits. Spatiotemporal expression patterns of the genes encoding the subunits are regulated by developmental and metabolic signals. The expression of large subunit genes in different organs might lead to a variation in the regulatory properties of AGPase [16]. 

**Figure 2:**
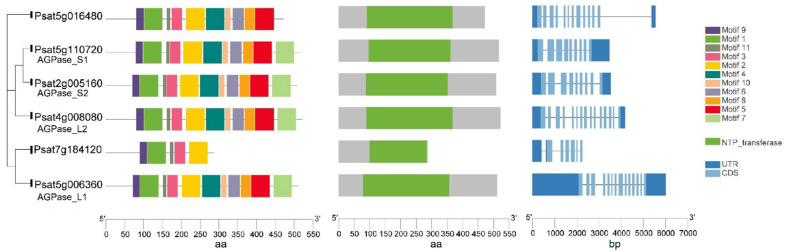
Gene structure, functional domains and conserved motifs in putative ADP-Glucose-Pyrophosphorylase (AGPase) subunits in peas. All subunits contain an NTP_transferase domain.

A mutation at the *rb* locus in peas results in the absence of one of the polypeptides of AGPase and consequently leads to a 10- to 40-fold reduction in AGPase activity during embryo development (Table 1). The mutation also has pleotropic effects on the seeds including a seed shape change from round to wrinkled, a starch content reduction of 50%, and an increase in lipid and sucrose content [17]. *Psagps1* and *Psagps2* encode the smaller subunits of AGPase, while *Psagpl1* encodes the larger subunit in peas. Experimental data have shown that the genes encoding smaller subunits are selectively expressed [18]. *Psagps1* transcripts are found in all tissues, with the highest level in developing seeds. In contrast, *Psagps2* is expressed mainly in seeds but at a lower level compared with *Psagps1*. *Psagpl1* is more selectively expressed than either of the small subunits with a high expression in sink organs (seed, pod, and seed coat) and it is undetectable in leaves. The pea lines with RNAi-mediated repression of the small subunit *Psagps2* produced wrinkled pea seeds with a starch content reduction of 40–50% and 25% less amylose. The repression of *Psagps2* caused the re-partitioning of carbon into other pathways and activated amino acid and storage protein synthesis. These complicated responses, including hormonal, metabolic and transcriptional changes, ultimately influenced seed metabolism and morphology [19]. DNA and amino acid sequences of two putative large subunits, AGPase_L1 (*Psat5g006360*) and AGPase_L2 (*Psat4g008080*), and two small subunits, AGPase_S1 (*Psat5g110720*) and AGPase_S2 (*Psat2g005160*), were retrieved from the reference genome assembly of the pea cultivar Caméor (https://urgi.versailles.inra.fr/Species/Pisum/Pea-Genome-project (accessed on 31 July 2021)). All of these AGPase subunits share a common core region—the NTP_transferase (nucleotidyl transferase) domain - that is indispensable for catalytic activity. In addition, they all share 11 conserved motifs. Although the DNA sequence of the two large subunits is longer than that of the small subunits, the length of the amino acid sequence is similar among all subunits. Another two genes (*Psat5g016480* and *Psat7g184120*) share high similarities to AGPase subunits at the amino acid level. The predicted protein of *Psat5g016480* contains an NTP_transferase domain and has 10 out of 11 conserved motifs. It is likely that the annotated *Psat7g184120* does not represent the full-length gene and that an incomplete NTP_transferase domain was identified in this gene (Figure 2).

SS catalyzes the transfer of a glucose (Glc) unit from ADP-Glc to the non-reducing end of a growing glucan chain of amylose or amylopectin through the formation of a new α-1,4-glycosidic linkage. SSs have multiple isoforms with differential organ specificity as well as temporal regulation, and can be categorized into GBSS and soluble SS. Each individual isoform plays a unique role in the biosynthesis of amylose and amylopectin. GBSS is required for amylose biosynthesis within the granule matrix (for a review of amylose biosynthesis, see [28]), while soluble SSs are primarily responsible for amylopectin biosynthesis [29]. 

GBSS is the most abundant protein found within starch granules. Multiple isoforms of GBSS have been found in pea seeds, rice and maize endosperms and Arabidopsis leaves [28]. GBSS is distinct from the other SS isoforms in its exclusive localization within the granule matrix and in its mode of action. GBSS adds glucosyl units processively from ADP-Glc to a malto-oligosaccharide (MOS) and does not necessarily dissociate from its product. Instead, it adds more glucosyl units to the product to build up a linear chain of substantial length [30]. In contrast, other SS isoforms act in a distributive way on MOS by dissociating from their product after the addition of a single glucosyl unit from ADP-Glc [31]. GBSS catalytic activity is stimulated by the presence of MOS [32] and regulated by protein phosphorylation [33]. 

GBSS is largely involved in amylose synthesis via the processive mode of action, leading to a linear polymer of up to the degree of polymerization (DP) 6000. Furthermore, GBSS plays a role in amylopectin synthesis by extending the existing side chains of amylopectin and contributing to the formation of long glucans (extra-long chains) of amylopectin [11,30,34]. However, it remains largely unknown what factors determine the partitioning of GBSS activity between these two processes. In the isolated starch granules from the developing pea embryos, GBSS incorporates the glucose into amylopectin rather than amylose. One possible explanation is that specific factors or conditions, such as small soluble glucans, are required for amylose synthesis that are lost during the isolation of starch granules [32]. This suggestion is supported by the evidence from experiments in which higher rates of incorporation of glucose into amylose, relative to amylopectin, are observed when starch granules are prepared without extensive washing. Further support comes from the finding that amylose synthesis is greatly stimulated when pure samples of MOS with less than eight glucose units are added to the isolated granules [35].

Several duplications of GBSS during plant evolution have led to tissue-specific and biochemical specialization and diversity among paralogues [28]. Two isoforms of GBSSI have been identified in peas: GBSSIa and GBSSIb. GBSSIa is expressed predominantly in embryos and involved in the synthesis of storage starch [20], while GBSSIb is predominantly found in leaves and contributes to the formation of transitory starch. The isoforms of GBSSIa and GBSSIb have distinctive properties despite the high structural and amino acid sequence similarities between the two proteins. This is mirrored by the distinct forms of amylose with different functional behaviors in different tissues of pea [36]. GBSSII is highly expressed earlier in embryo development than GBSSI [20]. Apart from being granule-bound, GBSSII accounts for 60-70% of the soluble SS activity in embryos. Although it remains unclear if GBSSII is involved in amylose biosynthesis, the evidence has indicated that GBSSII alone cannot synthesize amylose. Alternatively, GBSSII may have a separate role in glucan formation other than amylose synthesis within the starch granule [37]. The differential expression of GBSS isoforms is also a common phenomenon in cereals. Wheat GBSSI is primarily found in the endosperm, while the other isoform, GBSSII, is expressed in leaves, pericarp, and aleurone [38]. 

The mutants and transgenic lines of many plant species with defective GBSSI have low amylose or have amylose-free (or waxy) starch [35]. The low amylose pea mutant lines (*lam*) lack or contain a greatly reduced amount of GBSSI (Table 1). The lack of GBSSI activity cannot be compensated for by GBSSII, suggesting that they have distinct mechanisms by which they contribute to amylose synthesis, and GBSSI has the unique ability to elongate MOS to make long chains of amylose [6,37]. The suggested unique property of GBSSI is its specific interaction with amylopectin in the matrix of the starch granule, which allows it to make long, linear polymers while other isoforms cannot [6]. GBSSI is also involved in the synthesis of amylopectin with super long (or extra-long) chains that have similar lengths as amylose and resemble the branched amylose with a few long branches [39]. As well as being a substrate for GBSSI, amylopectin behaves as an effector for GBSSI to further drive amylose synthesis forward. In the presence of amylopectin, GBSSI has a stronger affinity to MOS as a substrate [40]. 

Soluble SSs are incorporated within stromal and granular fractions found in the granule matrix as granule-associated proteins [41]. Soluble SSs can be sub-divided into starch synthase I (SSI), starch synthase II (SSII), starch synthase III (SSIII) and starch synthase IV (SSIV). More recently, a new class of SS named SSV was identified in plants that are most closely related in sequence to SSIV and might result from a gene duplication event [7,42,43,44]. SSV appears to be quite different from other SSs with a non-enzymatic role in starch granule initiation in Arabidopsis [7]. The functional characterization in more species is required to better understand its role in starch biosynthesis. Each SS class has a distinct role in amylopectin synthesis, albeit with a functional overlap in some instances [45]. SSI elongates the shortest MOS/α-glucan chains and SSII further elongates the intermediate glucan chains produced by SSI. In addition to its role in granule initiation and hilum formation, SSIII synthesizes the longest linear cluster-spanning B chains in amylopectin of DP >30 by elongating the glucan chains produced by SSII [1,39]. SSI, SSII and SSIII are directly involved in amylopectin biosynthesis via the catalytic transfer of a Glc unit from ADP-Glc to a growing glucan chain through the formation of α-1,4-glucosidic linkage. The resultant glucan chains of varying lengths can be further elongated, branched (by starch branching enzymes) or de-branched (by starch debranching enzymes) [39]. In contrast, some evidence suggests that SSIV is less involved in amylopectin biosynthesis but plays a unique role in starch granule initiation by interacting with non-catalytic proteins, as well as in the control of granule morphology and the degree of starch accumulation [1,39,46]. Previous studies indicate that soluble SSs have differential spatial expression patterns in peas. SSII is the major soluble isoform in pea embryos while SSIII is the dominant soluble SS isoform in leaves, although a small proportion of starch synthase activity can be attributed to SSII in the latter. Similarly, the SSIII-like isoform can be detected in pea embryos [41]. Up to 20% of the measurable starch synthase activity of starch granules from pea embryos is attributable to SSII [6]. The mutation of the SSII gene at the *rug5* locus in pea results in abnormal starch granule morphology and amylopectin structure with fewer chains of intermediate length and more very short and very long chains [27] (Table 1).

All SS isoforms are structurally related; they share a highly conserved C-terminal catalytic domain but have a variable N-terminal extension. It is suggested that the various functions of each SS class in different tissues and between species account to some extent for the structural variations between starches from different botanical sources. Gene duplications have diverged into the different isoforms of some SS classes in cereals [1]. These isoforms have a high degree of similarity in protein sequences but are often differentially expressed in tissues such as in the endosperm or vegetative tissues. The “a” isoforms of SSII and SSIII appear to be predominant in the cereal endosperm, based on gene expression and the mutant studies. In other plant species having storage starch-filled organs (e.g., potato and pea), there is only one isoform for each class. However, it is still not fully understood how each class fulfils its proposed role at the molecular level [1]. 

**Figure 3:**
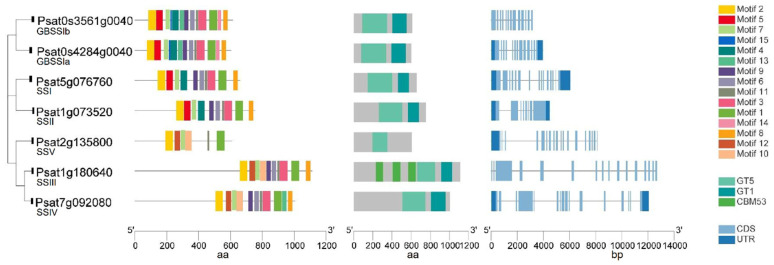
Gene structure, functional domains and conserved motifs in putative granule-bound starch synthase (GBSS) and soluble starch synthase (SS) isoforms in peas. Domains identified are carbohydrate-binding modules of family 53 (CBM53) domain, glycosyltransferase-5 (GT5) domain and glycosyltransferase-1 (GT1) domain.

Putative genes encoding GBSS and SSI–SSV in pea were identified by a blast search against the pea reference genome sequence database (Figure 3). Similar to the SSs in other species, GBSS has a very highly conserved protein sequence, and more variability is present in the N-terminal of soluble SSs in peas. Both GBSSIa (*Psat0s4284g0040*) and GBSSIb (*Psat0s3561g0040*) have a glycosyltransferase-5 (GT5) domain, a glycosyltransferase-1 (GT1) domain and 13 conserved motifs with a similar distribution. Each soluble SS has a conserved domain of GT5 and GT1 except that SSIII and SSV each has only GT5. Similarly, in maize, SSV has a domain of GT5 but not GT1 [44]. The putative SSIII (*Psat1g180640*) is the longest isoform with three carbohydrate binding modules (CBMs) of family 53 (CBM53) domains located in tandem in its N-terminal that are not present in other SS isoforms. CBMs are non-catalytic domains with carbohydrate-binding sites connected to catalytic domains, which promote the affinity of the enzyme (catalytic domains) with the substrates by increasing the concentration of the substrate on the surface of the enzyme [47]. SSIII in other plant species also contains three CBM domains in tandem [48]. One such example is that three CBM53 domains in the SSIII isoform in Arabidopsis have starch-binding activities and modulate the catalytic properties of the enzyme [49]. In maize, the SSIII isoform has three CBM25 domains in tandem in its N-terminal [44].

**Figure 4:**
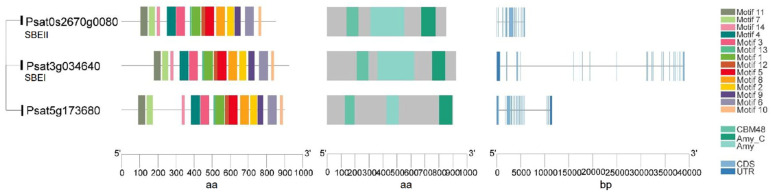
Gene structure, functional domains and conserved motifs in putative starch branching enzyme (SBE) isoforms in pea. Domains identified are the carbohydrate-binding module family 48 (CBM48) domain, α-amylase (Amy) domain and α-amylase C-terminal (Amy_C) domain.

Starch-branching enzymes (SBEs) are structurally related to the α-amylase superfamily of enzymes and determine the starch polymer structure. SBEs introduce α-1,6-branch points in glucan chains through the cleavage of internal α-1,4 bonds and the transfer of the released reducing ends to C-6 hydroxyls, and produce new non-reducing ends for glucan elongation by SS and starch phosphorylase [50]. SBEs stimulate the activity of SS and they act together and sequentially during the biosynthesis of amylopectin [21,50]. Higher plants such as maize, rice and peas have multiple isoforms of SBEs that can be divided into two categories: SBEI and SBEII. The coordinated actions of multiple SBEs are the determinants of the branching pattern and chain length distribution (CLD) in amylopectin. SBEI and SBEII are representatives of two different families of branching enzymes with distinct biochemical properties and different preferences for the glucan substrate in pea seeds. It is suggested that SBEI from pea and SBEII from maize belong to the same SBE family, while SBEII from pea and SBEI from maize form another category [21]. A transposon-like insertion at the *r* (*rugosus*) locus led to the complete loss of SBEI activity in the developing pea embryos producing the wrinkled (*r*) phenotype, one of the traits studied by Mendel in peas [22]. SBEI contributes to the synthesis of about 75% of the amylopectin in mature pea embryos and creates a polymer that is less soluble than the polymer created by SBEII. SBEII may be more successful in catalyzing the synthesis of shorter chains. The genes encoding these two isoforms are differentially expressed during pea embryo development, with SBEI highly expressed in young embryos and SBEII seen as more abundant in later development [21]. Wrinkled pea (*r*) seeds have a reduced total starch and amylopectin content compared to round peas, as well as an altered starch granule morphology [21]. This indicates that SBEI can limit the rate of starch synthesis and that, during early seed development stage, the contribution of SBEI is essential to the formation of normal starch granules and cannot be compensated for by other isoforms. In addition to the profound changes observed for starch, *r* mutant seeds also show pleiotropic effects and complex metabolic fluctuations such as a higher level of free sucrose, more lipid, less legumin and a reduced seed longevity [22,23]. No mutation in the pea SBEII gene has been identified through the screening for wrinkled seeds, suggesting that the mutations of SBEII do not lead to the wrinkled phenotype. This might be the result of a smaller contribution of SBEII to amylopectin biosynthesis and its later activity during embryo development [21]. In peas, both SBEI (*Psat3g034640*) and SBEII (*Psat0s2670g0080*) have a conserved CBM48 domain at N-terminal, an α-amylase (Amy) domain in the center, and an α-amylase_C (Amy_C) domain that are signature domains of SBE (Figure 4). Of note, although SBEI and SBEII have similar lengths of amino acid sequence, SBEI has an extremely long DNA sequence as a result of multiple long introns. Psat5g173680 has a high sequence similarity to SBEI and SBEII, and needs to be characterized to determine whether it is an SBE isoform.

Starch debranching enzymes (DBEs) hydrolyze the α-1,6-glucan branches of amylopectin and these processes are required for the normal synthesis of amylopectin by trimming excess branches [29]. These enzymes belong to the glycoside hydrolase family 13 (GH13) and share the catalytic Amy domain and a CBM48 with SBEs. There are two classes of debranching enzyme, isoamylase (ISA) and pullulanase (PUL) (also known as R-enzyme or limit-dextrinase, LDA), with differences in their amino acid sequences and substrate preferences [51]. Although there is some functional overlap between DBEs [52], ISAs differ from PUL in their inability to hydrolyze pullulan and their limited action on α-limit-dextrins [53]. Three ISAs–ISA1, ISA2 and ISA3, and one PUL have been reported in plants [1]. ISA1 and ISA2 are involved in the determination of the glucan branching pattern and growth of starch granules. ISA removes the irregularly spaced branches in pre-amylopectin so that only the appropriate chains are elongated by SSs to facilitate or accelerate the crystallization of glucans [54,55]. Debranching by ISA is a mandatory step, but phenotypes of mutants lacking the enzyme suggest that this is not strictly essential for making a crystallization-competent glucan. Mutation of either of these genes leads to a decreased concentration of amylopectin and a concomitant accumulation of soluble phytoglycogen, even though some insoluble starch is still made [56,57,58,59]. Low amylopectin content caused by the lack of debranching enzyme activity implies that it plays a significant role in determining the fine structure of amylopectin, although the exact mechanism remains unknown. In addition to their function in starch synthesis, ISA3 and PUL primarily debranch starch during its degradation [51,52,60]. In Arabidopsis leaves, ISA3 shows partial redundancy with ISA1 for starch synthesis and PUL has dual functions that are partially redundant to ISA3 for degradation and to ISA1 for synthesis [52]. Similarly, PUL functions during starch synthesis as well as degradation of granules in maize endosperm [51]. 

ISA generally functions as a multimeric enzyme in heterometric or homometric complexes depending on the species, and consists of up to six composite peptides. In the dicots such as Arabidopsis, AtISA1 and AtISA2 form a heteromultimetric enzyme and both are required to be present for catalytic activity [61]. Similarly, in potato tubers, Stisa1 and Stisa2 are associated as a multimeric enzyme and act together to debranch soluble glucan during starch synthesis [58]. A heteromeric complex containing ISA1 and ISA2 was also reported in developing kidney bean (*Phaseolus vulgaris*) seeds [62]. However, in monocots such as maize, both homomeric ISA1 and heteromeric ISA1/ISA2 complexes were identified in the endosperm and either of them is sufficient for near-normal starch synthesis [63]. 

**Figure 5:**
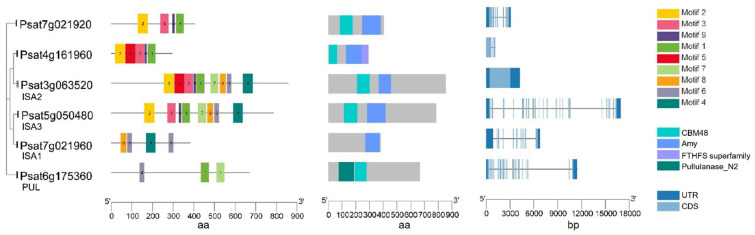
Gene structure, functional domains and conserved motifs in putative starch de-branching enzyme isoamylase (ISA) isoforms and pullulanase (PUL) in pea. Domains identified are carbohydrate-binding module family 48 (CBM48) domain, α-amylase (Amy) domain, FTHFS superfamily domain and Pullulanase_N2 domain.

Both types of debranching enzymes were identified in peas, suggesting their roles in starch metabolism in the developing embryo and in starch degradation during germination [64]. Three genes encoding the different isoforms of ISA (Psisa1, Psisa2 and Psisa3) were identified in peas. All three isoforms are able to bind to glucan substrate although Psisa2 lacks catalytic ability [53]. Similarly, in potato, Stisa2 encoding an ISA shows no discernible catalytic activity on its own, while it may retain the ability to bind glucan [58]. In pea, all the putative DBEs except ISA1 have a CBM48 domain (Figure 5) and all three ISAs have an Amy domain that is not present in PUL. PUL has a pullulanase_N2 domain that makes it distinct from other ISAs. Another two genes (*psat7g021920* and *Psat4g161960*) with a CBM48 domain and an Amy domain are also identified. Further studies are required to investigate their possible functions for a better understanding of whether they play a role in starch biosynthesis.

### 2.2. Other Starch Metabolism-Related Enzymes 

Mutations at the *rug4* locus result in the loss of most of the activity of Susy in the developing embryos of peas (Table 1). The *rug4* mutants have a wrinkled seed phenotype and reduced starch content in the embryos [25]. Genes encoding three isoforms of Susy (*SuSy1*, *SuSy2*, and *SuSy3*) have been isolated from peas. These isoforms have distinct expression patterns and kinetic properties. The mutant line *rug4-c* carrying a mutation at *SuSy1* has only 5% of SuSy activity in the embryo, but with minimal pleiotropic effects on other enzymes in primary metabolism. The starch content of the *SuSy1* embryo is reduced by 30%, while the cellulose content is unaffected. This finding suggests that SuSy1 in embryos is necessary for starch synthesis but not required for cellulose synthesis, and that different isoforms of SuSy may have distinct roles in carbon channeling [26]. These studies further demonstrate that the alternative degradation pathway for sucrose via invertase (INV) cannot compensate for the lack of SuSy activity in pea [10]. In agreement with this finding, the heterologous expression of a yeast INV in the embryos of *Vicia narbonensis*, a close relative of peas, leads to an approximate starch reduction of 50% in cotyledons [65]. While in cereals, once sucrose has been translocated to sink tissues, it is exported and catabolized by either SuSy or INV to provide carbon skeletons for growth and metabolism [8]. Studies suggest that INV may play this role particularly in the early stages of endosperm development [30].

Phosphoglucomutase (PGM) catalyzes the interconversion of Glc-1-P and Glc-6-P in the cytosol and plastids. The pea *rug3* locus encodes a plastidial PGM. Five pea mutants at the *rug3* locus exhibit a wrinkled phenotype, reduced amylose content, and a starch content of between 1% and 20% of the dry weight compared with 50% in the wild type (Table 1). In pea embryos, Glc-6-P is imported from the cytosol into the amyloplast where Glc-6-P is reconverted to Glc-1-P by the plastidial isoform of PGM, providing the substrate for the committed step of starch biosynthesis [24]. Similarly, in potato tubers, plastidial PGM is involved in starch biosynthesis. The transgenic inhibition of the *StpPGM* gene in potato tubers leads to a significant reduction in plastidial PGM activity. The tubers from the transgenic lines show up to a 40% decrease in starch, and significant increases in the levels of sucrose and hexose phosphates [66]. 

Starch phosphorylase (SP), also called α-glucan phosphorylase, catalyzes the reversible transfer of glucosyl units from Glc-1-P to the non-reducing end of α-1,4-glucan chains with the release of phosphate. It may be involved in either synthetic or degradative reactions depending on the relative concentrations of the soluble substrates. Two distinct forms of SP, plastidial and cytosolic, exist in higher plants [30,67]. Mounting evidence suggests that plastidial SP is involved in starch biosynthesis in storage organs although the precise mechanism is unclear [68,69,70]. The loss of plastidial phosphorylase (Pho1) results in a significant decrease in starch content and the accumulation of smaller starch granules with a modified amylopectin structure in rice seeds. Pho1 is thought to be involved in the glucan initiation process by synthesizing glucan primers with a long DP [70]. Two Pho isoforms, Pho1 and Pho2, were identified in pea cotyledons with distinct expression patterns and subcellular locations. The increased expression of cytosolic Pho1 was observed during seed germination, while plastidial Pho2 was predominant in the developing seeds. Therefore, it is argued that Pho2 is more likely to play an important role in the process of starch granule formation [68]. Previous studies suggest that SP also catalyzes the phosphorolytic degradation, although its precise mechanism remains unclear [67].

CBMs are a type of non-catalytic structural domain of carbohydrate-active enzymes with carbohydrate-binding activity. They promote the association of the enzyme (catalytic domain) with the substrates by bringing the enzymes into close and prolonged proximity with their substrates [47,71]. CBMs can be grouped into 88 families based on the similarity of amino acids sequences (http://www.cazy.org/Carbohydrate-Binding-Modules.html (accessed on 31 July 2021)) with variable substrate specificity and biological functions. Arabidopsis Protein Targeting to Starch 1 (AtPTST1) and AtPTST2 contain a CBM48 domain. AtPTST1 regulates amylose biosynthesis in leaves by mediating the binding of GBSS to the developing starch granules. Arabidopsis *ptst* mutants demonstrate a dramatic reduction in GBSS proteins in starch granules which leads to the production of amylose-free starch and a similar phenotype to other mutants lacking GBSS [72]. AtPTST2 facilitates granule initiation by delivering suitable MOS substrates to SS4 and influences the morphology of the transient starch in leaves [73]. OsGBP, a homolog of AtPTST1 in rice, participates in both leaf and endosperm starch biosynthesis by mediating the binding of GBSS proteins to the developing starch granules [74]. 

## 3. Post-Translational Protein Modifications and Coordinated Multi-Enzyme Complexes

Starch biosynthesis is a coordinated and complex process with many core enzymes and non-enzyme proteins involved. Many of these enzymes are regulated by different post-translational modifications and control mechanisms [39]. Accumulating evidence suggests that the phosphorylation of these enzymes, and the formation of complexes between starch biosynthetic enzymes, are required for starch biosynthesis. Protein phosphorylation activates the catalytic activities of SBEIIa and SBEIIb, but not SBEI, in amyloplasts of wheat endosperms [75], and may also regulate their association with starch granules [33]. The phosphorylation of GBSSI was observed in starch granules of maize endosperms [33]. The oligomerization of GBSSI in rice endosperms was promoted by post-translational protein phosphorylation [76]. Wheat amyloplast SBEIIb and SP each co-immunoprecipitated with SBEI in a phosphorylation-dependent manner, suggesting that the phosphorylation of one or more of these proteins might be a prerequisite for enzyme complex formation [75]. Protein phosphorylation also plays a critical role in the association of SS-SBE in a multi-enzyme protein complex. The phosphorylation-dependent complexes of SSI, SSIIa, and of either SBEIIa or SBEIIb, were observed in wheat endosperm amyloplasts [77]. Similarly, in maize endosperms, SBEIIa, SBEIIb, and SSIIa co-immunoprecipitated with SSIII in a phosphorylation-dependent manner. In maize, another smaller complex was also observed among SSIIa, SBEIIb, and SBEIIa, but lacked SSIII [78]. However, monomeric forms of all four proteins were also detected in the developing maize endosperms [79]. SSIIIa regulates resistant starch content in rice depending on the high expression of the *Waxy^a^* (*Wx^a^*) allele, and their interaction at the post-translational level might be part of a network of starch biosynthetic enzymes [80]. These observations highlight the inherent complexity of the tight coordination and phosphorylation of multi-enzyme complexes required for starch metabolism.

Post-translational redox modulation also regulates starch synthesis in plants. Several enzymes involved in transient starch metabolism in photosynthetic tissues are sensitive to redox modulation, although in vivo evidence is still lacking for most of them [81]. Much fewer studies have been carried out on the influence of such post-translational processes on storage starch accumulation. The intermolecular reversible disulfide bond formation between the cysteine residues of the two small units induced by redox modification leads to changes in AGPase activity in potato tubers [82]. As more evidence emerges for the relevance of the redox sensitivity of starch metabolic enzymes, altering the redox regulation of certain enzymes may open up potentially new avenues for tailoring starch biosynthesis to improve yield and functionality to meet industry requirements.

It appears that the formation of the complexes of SS and SBEII are conserved within cereal endosperms and that they may function in the global regulation of starch biosynthesis and granule formation [1], although the precise function of various enzyme complexes is not fully understood. One such example is that the coordinated enzyme sets of GBSS, SBE and SS control the biosynthesis of both amylose and amylopectin in rice [83]. Likewise, the absence of one protein can affect the granule association of other biosynthetic enzymes in maize endosperms [33]. One possible explanation is that the enzyme complex might facilitate channeling the substrates from one enzyme to another and increase the overall efficiency of the process. The glucan chains elongated by SSs are the substrates of SBEs, and the branches created by SBEs can be directly elongated by SSs. The complex possibly confers enzyme specificity, optimizes the ratio of SS/SBE, and has effects on substrate binding, chain length and branching position and thus, specific, architectural consequences on the amylopectin structure and formation of semi-crystalline starch granules [1,30,79]. Another possible role of the complex is that it could protect the growing polymer from degrading enzymes that are also present within the amyloplasts [30]. 

## 4. Transcriptional Regulation Network of Starch Metabolism

In addition to the genes in the starch biosynthetic pathway, other genes involved in seed development, cellular metabolic flux and carbon partitioning also play a key role in starch synthesis. For example, the loss-of-function of 6-phosphogluconate dehydrogenase (6PGDH) in the oxidative pentose phosphate pathway causes a severely defective kernel with a reduced starch accumulation in maize [84]. The mutation of the pyruvate kinase gene, *OsPK2*, significantly reduces grain weight and starch content, and alters starch physicochemical properties in rice [85]. Recent evidence shows that auxin plays an important role in controlling starch accumulation in pea seeds [86]. The auxin deficiency mutant *tar2* (*tryptophan aminotransferase related2*) produces small, wrinkled seeds with a reduced starch content. The activity of different starch synthesis enzymes and the expression of the corresponding genes are reduced in the mutants, indicating that auxin is required for starch accumulation in peas. A link between auxin and the signaling sugar trehalose 6-phosphate (T6P) has been identified recently, in which T6P acts as an upstream positive regulator of TAR2 and auxin to facilitate the sucrose-to-starch conversion [87]. T6P regulates starch enzymes not only by feedback inhibition directly at the protein level [88], but also in the gene expression level in association with auxin.

A number of transcription factors (TFs) have recently been identified as regulators of some of the major genes involved in starch biosynthetic pathway in the endosperms of cereal crops. A basic leucine zipper transcription factor (OsbZIP58) in rice positively regulates starch content and composition, and the amylopectin CLD. OsbZIP58 binds directly to the promoters of and regulates the expression of six starch biosynthetic genes: *OsAGPL3*, *Wx*, *OsSSIIa*, *SBE1*, *OsBEIIb*, and *ISA2* [89]. Similarly, a novel bZIP family TF TabZIP28 is a transcriptional activator of starch synthesis that can bind to the promoter of cytosolic AGPase and enhance its transcription and activity in common wheat [90]. Moreover, some other TF families are reported to regulate starch synthesis. For example, the endosperm-specific TFs opaque2 (O2) and the prolamine box binding factor (PBF) are positive regulators of starch biosynthesis in maize [91]. The transcription factor TaMYB44 can bind to the promoters of *TaSUT1*, *TaSSIIIa*, *TaBEIIa*, *TaISA1*, and *TaBEIIb* in yeast, and is thought to be a regulator of starch biosynthesis in hexaploid wheat [92]. These reports highlight that TFs can positively regulate the expression of multiple starch biosynthesis genes. On the other hand, studies suggest some TFs are involved in the negative regulation of starch biosynthesis. A transcription factor, ZmEREB94, acts as a negative regulator of starch synthesis through regulating the expression of *Shrunken-2* (*sh2*), *Wx*, *SSI*, *BT1*, and *SBE1* in maize [93]. A NAC TF NAC019-A1 can bind to the promoter of the AGPase small subunit 1 and repress its expression, hence negatively regulating starch synthesis in wheat endosperms [94]. In addition, some experimental data provide evidence that some TFs regulate the accumulation of both starch and storage proteins. Two endosperm-specific NAC TFs in maize, ZmNAC128 and ZmNAC13, can coordinate the accumulation of starch and proteins through transcriptional regulation of key starch biosynthetic genes and the major seed protein genes [95]. An endosperm-specific TF TaNAC019 can simultaneously regulate glutenin and starch accumulation in wheat. It directly binds to and activates the expression of HMW-GS genes, the SS gene *TaSSIIa*, and the SuSy gene *TaSuSy1*. It also indirectly regulates the expression of *TaSPA*, a transcriptional regulator of genes encoding LMW-GS and gliadins [96]. 

Although previous efforts have been made to characterize the function of starch biosynthetic enzymes and to elucidate the process of starch biosynthesis in peas, it is important to note that the catalytic function of various isoforms of different enzyme classes have largely been deduced from mutant studies. However, single mutations can have pleiotropic effects since starch biosynthetic enzymes generally function as complexes. The pleiotropic phenotypes of the mutant might result from the disruption of enzyme complexes and their associations, and not just the mutated gene [30]. Such pleiotropy indicates that there might be more interactions between starch biosynthetic genes/enzymes and genes/enzymes involved in other signaling and metabolic pathways, highlighting that it is critically important to apply an integrated and network-based approach to obtain a more informative view of genetic mechanisms regulating starch metabolism. Sizable gaps still exist in our understanding of the dynamic molecular mechanism and transcriptional regulation network underlying starch biosynthesis and deposition during pea seed development. Furthermore, it is unclear how the differential gene expression patterns are associated with the variation of starch content, the ratio of amylose to amylopectin and thus the various functional properties of starch in different pea genotypes.

To the best of our knowledge, no studies in peas have reported on non-enzyme proteins such as CBM containing proteins, and the factors that can regulate starch synthesis through their interaction with starch biosynthetic enzymes. Very limited transcriptome research has been reported on the developing pea seeds. A comparative transcriptomics study was conducted on garden peas and grain peas at two developmental stages, 10 and 25 days after pollination (DAP) [97]. In this study, higher expression levels of several starch biosynthesis-related genes were observed in grain peas compared with garden peas. In addition, several TFs were differentially expressed at early and late stages of seed development between vegetable and grain peas. However, no TFs were identified that control the flux of carbon to starch and regulate the expression of starch biosynthetic genes during pea seed development. During the starch granule (number and size) growth in developing seeds, the characteristics of some enzymes and the relative importance of each enzyme are likely to change [3]. Therefore, it is essential to perform transcriptome profiling on pea seeds with varying starch content, the ratio of amylose to amylopectin, and/or the functional properties at different developmental stages, providing data that unveil the dynamic regulatory and co-expression networks underlying starch metabolism. It remains unclear how biosynthesis and the accumulation of starch is coordinated with seed storage protein during pea seed development. As demonstrated in cereal crops, some TFs can coordinate the accumulation of starch and proteins by regulating the expression of key starch biosynthetic genes and major seed protein genes. The gene regulatory network could potentially enable the identification of the TFs that simultaneously regulate starch and protein accumulation and coordinate their balance. The identification of TFs and other regulators controlling starch metabolism, along with novel isoforms of starch biosynthetic enzymes which need to be characterized, will allow for the targeted modification of pea starch content, composition, granule morphology, and thus the functional properties for specific end-uses. 

## 5. Genetic Mapping of Starch Metabolism 

Starch content and composition are quantitative traits determined by multiple genes, and their expressions are environmentally dependent. Genetic mechanisms of starch metabolism have been well studied in major cereal crops, whereas little information is available in legume crops including peas. Quantitative trait locus (QTL) mapping and a genome-wide association study (GWAS) coupled with transcriptome profiling have been conducted in cereal crops to decipher the mechanisms regulating starch metabolism: starch content, composition, granule size, and functional properties [98,99,100,101,102,103,104,105,106,107,108,109,110]. In rice endosperms, different alleles at the waxy locus lead to various levels of GBSS and hence contribute to the variation of amylose content and starch properties [98,99,111]. Similarly, in barley, single nucleotide polymorphisms (SNPs) of GBSSI are associated with a variation in the amylose content of grain starch [103]. SNPs also contribute to the variations in amylose fine structure [112]. However, less effort has been made to identify the loci and candidate genes for starch-related traits in legume crops. Only a few QTL studies have been reported to identify QTL underpinning starch content and composition. Five and one QTL were identified in starch and amylose content, respectively, in the common bean (*Phaseolus vulgaris* L.) [113]. Nine QTL were identified in seed starch content in a population of recombinant inbred lines (RILs) derived from an interspecific cross of *Glycine max* and *Glycine soja*. However, all these QTL were detected only in a single environment, indicating the environmental effect on their expression [114]. One major QTL was detected explaining 12.3–13.8% of total variance of the seed starch content in mung bean (*Vigna radiata* L. Wilczek) [115]. 

A few attempts have been made to identify SNPs associated with seed starch and amylose content, and amylopectin CLD in peas. Ten genes, including *AGPase_L1/S1/S2*, *GBSSI/II*, *SuSy2/3*, *SBEII*, sucrose phosphate synthase (*SPS*), and Glc-6-P/phosphate-translocator, involved in starch metabolism, were amplified from fifty diverse *Pisum sativum* accessions. Four SNPs with one within each of the genes *AGPase_L1* and *GBSSI,* and two within *SBEII*, were significantly associated with amylose concentration in two out of eight environments. Ten SNPs within the genes *SBEII*, *SuSy2* and *Sps* were significantly associated with the total starch concentration in at least five out of eight environments [116]. Similarly, partial sequences of 25 candidate genes representing 16 enzymes involved in starch metabolism (including genes responsible for the substrate supply, glucan chain elongation, branching, debranching, phosphorylation and degradation) were characterized for polymorphisms in a panel of 92 diverse pea accessions. Association mapping was applied to identify the gene polymorphism associated with amylose content and CLD. Seven out of 25 candidate genes were significantly associated with CLD. Of these seven significant candidate genes, three genes (*r*, *UGPase*, *AGPS2*) showed a significant association with CLD in both greenhouse and field conditions. Not surprisingly, amylose content was associated with the *r* locus in both environments [117]. In addition to candidate genes association analysis in the aforementioned studies, linkage mapping for starch content was conducted in two pea RIL populations PR-02 and PR-07 [118]. QTL were detected for the starch content on LG2b and LG4a in population PR-02, with QTL on LG2b detected in two out of the three trials. QTL on LG1a, LG3b, LG3c and LG7a for starch content were identified in repeated trials in the population PR-07. No common QTL were detected in these two populations, although they shared one common parent, CDC Striker, as the pollen donor. More recently, GWAS was performed for seed starch content in 135 pea accessions collected from 23 different global breeding programs [119]. Nine SNP markers located on four chromosomes (LG2, 3, 5 and 7) and one scaffold were significantly associated with starch content in three or more environments. Further characterization of these QTL and identification of novel QTL will allow for a better understanding of the genetic mechanism that governs the starch metabolism in pea seeds. 

## 6. Genetic Resources and Genomics of *Pisum*

All *Pisum* species are diploid (2*n* = 14) with a genome size of about 4.45 Gb in the recently sequenced *Pisum sativum* L. cv. Caméor, although the variation in genome size across the genus remains unknown [120]. *Pisum* has two or three distinguished species. The *P. sativum* complex, including cultivated *P. sativum* subsp. *sativum* and wild subsp. *elatius* Asch. & Graebn. which is native to the European-Mediterranean region and mid-northwest Asia, while *P. fulvum* Sibeth. & Sm. is only found in the Middle East and is distinguished from *P. sativum* by crossing barriers, dehiscent pods and seeds dormancy [121,122]. *P. sativum* subsp. *abyssinicum* A. Br. is cultivated in Ethiopia and Yemen and has no wild counterpart. Compared with other subspecies, *P. sativum* subsp. *abyssinicum* A. Br. possesses several unique traits such as strongly serrate leaflets, a glossy seed coat and early flowering [123,124,125]. Phylogenetic studies found that *P. fulvum* and *P. sativum* subsp. *abyssinicum* form sister clades. *P. sativum* subsp. *elatius* falls between *P. fulvum*–*P. sativum* subsp. *abyssinicum* and cultivated *P. sativum* [123].

Several pea germplasm collections have been assembled and curated worldwide. Currently, the largest pea germplasm collections are held by INRAE France (8839 accessions and over 9000 lines of TILLING mutants, http://florilege.arcadproject.org/fr/crb/proteagineux/crb-proteagineux, http://urgv.evry.inra.fr/UTILLdb (accessed on 31 July 2021)) [125]. In addition to wild and cultivated accessions, there are also pea mutant stocks held at several research centers [123]. The U.S. National Plant Germplasm System (NPGS) repository at the US Department of Agriculture (USDA) (https://npgsweb.ars-grin.gov/gringlobal/descriptors (accessed on 31 July 2021)) holds 6827 pea accessions. The adoption of the Germplasm Resource Information Network (GRIN-Global) database by the USDA allows for online queries across multiple traits, including phenological and morphological traits; seed mineral nutrient composition and protein concentration; and biotic stress resistance. The USDA pea core collection of 504 accessions was assembled based on geographical origin and flower color, representing ~14% of all USDA pea accessions at the time of construction [126]. This core collection of pea lines, including cultivated and wild subspecies, represents a broad range of genetic variation and diverse phenotypic expression. Plant Gene Resources of Canada (PGRC) also has a diverse collection of worldwide pea germplasm including landraces and key Canadian varieties. The landraces contain much of the original diversity, accumulated mutations and historic recombinations present since domestication [123]. There is increasing interest in utilizing crop wild relatives and landraces to accelerate genetic gains for crop improvement. Although tremendous genetic diversity is present in the *Pisum* species, the diversity studies have mainly concentrated on cultivated subspecies [125]. The pea breeding targets have mainly focused on yield, disease and lodging resistance, visual seed quality traits and protein content. Some studies have demonstrated that pea wild relatives not only are potential sources of resistance to biotic stresses [127] and tolerance to abiotic stresses [128], but also show a positive effect on seed yield and yield components [129]. A *Pisum elatius* accession was identified as a double null mutant for two closely linked genes encoding the TI1 and TI2 seed protease inhibitors, demonstrating that pea wild relatives can be exploited for increasing the nutritional value of peas [130]. Despite starch accounting for 50% of pea seed dry weight, relatively limited effort has been made to characterize the diversity of starch-related characteristics and to elucidate the underlying genetic mechanisms of starch metabolism. The diverse pea germplasm can serve as the platform for genetic studies of seed nutritional quality traits, including the discovery and characterization of new genes and alleles involved in starch metabolism, which ultimately facilitate the development of new pea varieties with diverse starch functional properties.

The advances of next-generation sequencing (NGS) technology and, consequently, the significant reduction in the cost of sequencing enable the construction of pea pan-genomes for multiple reference genomes representing different subspecies. The multiple reference genomes will enable more accurate identification of structural variations and allow for the capturing of all possible functional variants which cannot be achieved by using a single reference genome. The availability of the first annotated reference genome assembly for a pea cultivar ‘Caméor’ [120] facilitates transcriptome assemblies, trait mapping using association and linkage studies, candidate gene predictions, molecular maker developments and other sequencing-enabled studies. As more genetic resources and genomic tools become available [118,120,131,132], this will accelerate closing the gaps in our understanding of genetic mechanisms controlling starch metabolism, and lead to the rapid development of novel pea cultivars with custom-designed starch.

## 7. Starch Granule, Functional Properties and Applications

Starch has a semi-crystalline granular structure consisting of amylose and amylopectin. Although the relative location of amylose and amylopectin in native starch granules is still under discussion, it is generally accepted that amylopectin is in the semi-crystalline structure, while amylose is in the amorphous regions of the amylopectin blocklets [39]. Starches from different botanical sources vary considerably in the proportion of amylose and amylopectin, granule morphology (size, shape and structure) and thus, physicochemical properties and functionality [133]. The CLD of amylopectin is a characteristic of the botanical origin of the starch [134]. An association exists between average chain length and crystalline allomorphs of starch granules [39]. However, the internal structures of granules are remarkably similar, consisting of growth rings, blocklets, and crystalline and amorphous lamellae [135]. A recent review [39] outlines the current understanding of starch granule architecture and the building block backbone model of starch structure. 

Starch granule size is an important factor for many applications and uniform granules are required for both food and non-food industrial applications [3]. One example is that starch granules of a uniform size are mixed with similarly sized encapsulated ink particles to produce carbonless copy paper [136]. The biodegradable plastic industry requires starch with small granules and a high amylose content [10]. However, the process governing the initiation of starch granules and the mechanisms that determine granule size, number, and morphology are still not well understood. These factors are under genetic control and are highly dependent on species and organs, which might result from the diversity in the functions of the proteins and enzymes involved. The mounting evidence suggests that plastid division plays an important role in determining granule size [137,138]. In addition, plastid tubule volume, cell size and shape contribute to the variation of granule size. Recent progress in understanding starch granule initiation and morphogenesis in Arabidopsis and cereal endosperms [139] will enable novel and improved biotechnological approaches to modify starch morphology and thus, tailor starch properties for various end-uses. 

A series of pea mutants have been used to characterize the changes of starch granule structure and the functionality as a result of the specific mutations in the starch biosynthetic genes. Four single mutations (*r*, *rug3-a*, *lam-c* and *rug5-a*) and the double mutation (*r*/*rb*) in pea all result in substantial changes in the amylose–amylopectin ratio and characteristic changes in granule structure and function [140]. The internal organization of amylopectin contributes to the type of crystal allomorph and stability of crystals in the granule [141]. The previous findings all suggest that amylopectin is the major building block of the basic starch granule architecture in wild-type plants [39]. This idea is reinforced by the discovery of amylose-free starch in wild-type plants, including cultivated crops and the model plant Arabidopsis [28]. Although the explanation for the precise biological role of amylose is long-awaited, available studies have suggested that amylose imparts the structural integrity of the granule to some extent, as waxy starch shows a reduced mechanical strength and is usually prone to cracking. While direct experimental evidence is currently lacking, it is speculated that amylose might provide some degree of plasticization of the amylopectin component [39].

In general, the starch from smooth peas has a high amylose content in a range of 33.1–49.6%, depending on the variety [142]. The granule size of pea starch ranges from 2 to 40 μm. Most of the granules are oval; however, spherical, round, elliptical and irregularly shaped granules are also observed. The crystalline structures of pea starch granules exhibit a C type that is intermediate between the A type (cereal starch) and the B type (tuber, root, high-amylose cereal starches and retrograded starches) [142,143]. Compared to smooth pea starch, wrinkled pea starch possesses a different amylose–amylopectin ratio and amylopectin CLD, and thus different thermal and functional properties. It contains 65% to 78% amylose and shows the B type crystalline pattern. The granule of wrinkled pea starch varies in size from 15 to 20 μm, displaying a lobed structure with a fusion of multiple small sub-granules into one single granule [144]. Amylopectin from isolated smooth pea starch (94.8% purity) contains a significantly smaller proportion of DP 6-12 short branch chains, but a larger proportion of DP 13-24 branch chains than those of maize and tapioca starches [145]. Pea starch has a high thermal stability and a relatively high resistance to mechanical shearing. It also possesses key functional attributes and provides some unique characteristics such as texturizing, gel formation, higher elasticity, and high concentrations of resistant starch greatly desired by the food industry [146,147]. However, a low pasting viscosity and a high extent of retrogradation due to the high amylose content are the major restraining factors for the widespread utilization of pea starch in the food industry [143].

Variations in the compositional and functional properties of pea starch have been reported in different pea genotypes/varieties. The variation in total starch and amylose content was observed in 50 diverse pea accessions grown in multiple environments [116]. Starch from various pea cultivars with different physicochemical and structural attributes resulted in significant variations in functional properties such as swelling power, gelatinization and pasting properties [148,149,150]. However, only a few genotypes were used in each of these studies for characterizing the functional properties of pea starch. Larger-scale systematic research is necessary to explore the relationship between the physicochemical and functional properties of pea starch. Very limited research has been directed towards exploiting the natural allelic variations available in diverse *Pisum* germplasms in order to modulate starch characteristics and, thus, functionality.

The successful application of starch depends largely on its two major functional properties: gelatinization and rheological properties including pasting properties, viscosity of starch paste, and rheological features of starch gel [151]. Various applications require different functional properties. Gelatinization is a process in which starch transforms from ordered semi-crystalline granules to an amorphous state resulting in granular swelling, crystallite melting, loss of birefringence, viscosity development, and solubilization [152]. Various techniques are used to study the gelatinization properties; differential scanning calorimetry (DSC) is the method most commonly used. Starch viscosity is a vital indicator of starch quality for both food and non-food applications. Viscosity measures the resistance of a fluid or semifluid to flow when a shear stress is applied [151]. The process of viscosity development is known as starch pasting. Peak viscosity is indicative of the water-binding capacity of the starch [152]. The ratio of amylose to amylopectin and the branch CLD of amylopectin are the major determinants of starch functionality [133]. There is a negative correlation between the peak viscosity and the amylose content of starch [145]. In addition, starch granule size is also a contributor to its functional properties [151]. 

Chemical (cross-linking, substitution, acid hydrolysis oxidation), physical (pre-gelatinization heat-moisture treatment, annealing), and enzymatic methods, or a combination of these methods, are currently applied to modify starch structure to improve the functional properties of pea starch [143]. Furthermore, pre-milling treatments such as thermal treatment (roasting and moist heating), partial germination and micronization have been attempted to modify pea flour/starch functional properties [153,154,155]. Apart from the large amount of water used, energy consumption and waste produced in these processes, and the increasing awareness of issues related to health and wellness by consumers, promote the use of more natural food ingredients and a shift away from chemical treatments. In addition to clean labeling, native pea starch with desirable functional properties and without modifications, offers more cost efficiency and sustainability for pea processing. This highlights the need for the development of pea varieties with diverse functional properties to expand the portfolio of pea starch targeting various specific applications. Pea starch with diverse functionality has a potential new market advantage and thus builds new value chains for the pea processing industry. 

Currently, most starch concentrates coming from pea fractionation are being used for food and animal feed, while there are increasing demands for pea starch to be used in non-food industrial applications. A comprehensive review was recently published on the industrial utilization of starches from pulse crops including peas [144]. Increasing environmental concerns about petroleum-based packaging materials require the development of environmentally friendly bio-based materials from renewable natural resources. Starch is used for making biodegradable packaging and edible films and coatings due to its abundant supply, low-cost, good processability and biodegradability [156]. Amylose is mainly responsible for the film-forming properties of starches and the properties of the films. The films made from linear amylose molecules are strong and flexible, while branched amylopectin-based films are weak and brittle [157]. In addition to better mechanical strength, the films prepared from high-amylose starches have better gas barrier properties [158]. Owing to its higher amylose content than many other natural starches, pea starch has been found to produce films with improved physical and mechanical properties [159]. Therefore, pea starch is a promising material for making biodegradable packaging and edible films. The bioplastic film made from pea starch presents excellent functional qualities such as strength, flexibility and elasticity, and shows the potential for use in food packaging and agricultural mulches (https://www.jic.ac.uk/research-impact/molecules-from-nature/impact/peas/the-history-of-pea-research-at-the-john-innes-centre/pea-starch-an-alternative-material-to-plastic-2/ (accessed on 31 July 2021)). Compared with conventional plastic polymers, the main drawbacks of starch-based biopolymers are hygroscopic and poor mechanical properties [160]. Some measures can be taken to mitigate these shortcomings, such as blending starch with other biodegradable biopolymers and the reinforcement with mineral, cellulose or starch nanocrystal fillers [161]. Pea starch films reinforced with waxy maize starch nanocrystals showed better mechanical, water-vapor barrier and morphological properties than pure pea starch film [160]. Pea starch-based composites reinforced with citric acid-modified pea starch showed improved storage moduli, glass transition temperature, tensile strength and water-vapor barrier capabilities [161]. Starch nanocrystals have been shown as good fillers to improve the mechanical and barrier properties of biocomposites [162]. Starch nanocrystals prepared from native pea starch granules by acid hydrolysis have been demonstrated as low-cost fillers showing greater potential to improve the PVA-based composite [156]. Nanoparticles are used as controllable drug release carriers to enhance the stability of drugs, thereby boosting the therapeutic efficacy and lowering drug toxicity and degradation [163]. These aforementioned examples highlight the potential applications for pea starch nanocrystals.

## 8. Future Prospects for Genetic Improvement of Pea Starch

The starch biosynthetic pathway is complicated and involves carbohydrate metabolism and seed development. Significant progress has been made in our understanding of starch metabolism in cereal endosperms; however, this does not necessarily equate with the metabolism in dicot embryos. Therefore, caution should be taken when attempting to infer the roles of particular isoforms in different species. Current genetic evidence demonstrating starch biosynthesis in peas is limited to mutant studies. Nevertheless, it is important to note that the interpretation of the function and contribution of an enzyme may be biased as starch biosynthetic enzymes form and act in coordinated complexes. As such, the pleiotropic phenotypes of the mutants might result from the combined effects of the disruption of multiple enzymes, as opposed to a single enzyme. Sizable gaps remain in our understanding of the genetic and molecular details of starch metabolism in peas. In particular, there is very limited knowledge about the transcriptional regulation of starch metabolic genes in pea. It is essential to identify genome-wide gene co-expression networks regulating dynamic starch biosynthesis at different seed development stages and to elucidate how the genes coordinate and interact to produce starch granules with specific composition and structure, and the mechanisms of complex interplay between starch and protein synthesis. Our knowledge of the isoforms of starch biosynthetic enzymes is not yet complete. The availability of a reference genome sequence and upcoming pan-genomes of pea facilitates the identification of more isoform candidate genes and novel non-enzyme proteins involved in starch metabolism. Together with subsequent functional genomics approaches, the integration of multiple ‘omics’-level datasets will provide the tools instrumental to elucidate starch metabolism in pea seeds. Furthermore, as the more efficient protocols become available for pea tissue culture and the regeneration of whole plants, genome editing technologies such as CRISPR/Cas9-based systems will enable high-precision molecular breeding in peas with tailored starch.

Over 65% of the output of a pea fractionation plant is a lower value product: starch. As new pea protein processors surface online, the volume of pea starch will dramatically grow at twice the rate of pea protein. Current uses for pea starch are limited to low-value commodity markets, mainly due to its less desirable and narrow range of functionality. The economics of a pea fractionation plant dictates creating higher-value starch opportunities (communications with industry). Therefore, major efforts are required to expand the functionality of pea starch for broad use in food and non-food industries. It is a necessary prerequisite for the value-added utilization of pea starch that more research is conducted to correlate composition, structure, and functional properties. Starch biosynthesis is a complicated biological process with multiple biosynthetic enzymes forming complexes. Therefore, the modulation of a single starch-synthetic gene may be insufficient to obtain starch with desirable functional properties. This highlights the need for applying an integrated systems approach to better understand starch metabolism at a more global level, to identify functional alleles in starch metabolic genes, and to characterize the extent to which the allelic variation contributes to the diversity of the functional properties of pea starch. The strategies that integrate genetic diversity, starch structure and functional properties have proved successful in the production of rice varieties with modulated starch characteristics, resulting in improved eating qualities [112]. Similar strategies have also been successfully demonstrated to expand the functional diversity of maize starch. The rich genetic resource of *Pisum* has tremendous untapped potential for the discovery of novel functional alleles in starch metabolic genes using GWAS, which will enable the identification of genetic variations that cannot be achieved by analyses of mutants. Such resources highlight the potential for diversifying pea starch composition, structure and thus, functional properties for applications of commercial significance.

Altogether, the research advances in the aforementioned aspects will enable the targeted breeding and molecular design pea varieties to produce novel starch with improved and/or customized functionality suited to specific industrial needs while maintaining a high protein content in pea seeds. This will ultimately promote the utilization of natural starch for food and non-food industrial applications. 

## Figures and Tables

**Table 1:** Overview of Pea Mutants with Defects in Starch Biosynthesis.

Mutant	Gene Mutated	Enzyme Function and Gene Expression	Mutant Phenotype	Reference
*lam*	Granule bound starch synthase Ia (*GBSSIa*)	Processively adds glucosyl units from ADP-Glc to its glucan substrate leading to a linear polymer–amylose and extends extra-long chains of amylopectin. Expressed in mature embryos where storage starch is synthesized.	Reduced activity of GBSSI and reduction of amylose content from 35% to about 8% compared with the wild type.	[20]
*r*	Starch branching enzyme I (*SBEI*)	Catalyzes the transfer of longer-branched chains and may be more involved in producing the more interior B-chains of the amylopectin. Expressed in early embryos. It contributes to the synthesis of about 75% of the amylopectin in mature pea embryos and creates a less soluble polymer. SBEI has the highest activity in branching amylose.	Wrinkled seeds with reduced total starch and amylopectin content, as well as altered starch granule morphology. Pleiotropic effects include a higher level of free sucrose and lipid, less storage protein legumin and reduced seed longevity.	[21,22,23]
*rb*	ADP-glucose pyrophosphorylase (*AGPase*)	Converts Glc-1-P and ATP to inorganic pyrophosphate and ADP-Glc. Both large and small subunits of AGPase are required for the synthesis of ADP-Glc.	Specific activity of the enzyme is 10 fold lower in the *rb* mutant embryos. Wrinkled seeds with starch content reduced from 50 to about 25% of the final dry weight, and sucrose and lipid contents increased from 5 to 9%.	[17,18]
*rug3*	Phosphoglucomutase [Plastidial] (*PGM*)	Reversibly converts Glc-6-P into Glc-1-P in the plastids and therefore provides the substrate for the committed step of starch biosynthesis—synthesis of ADP-Glc.	Wrinkled seeds with reduced amylose content, and a starch content of between 1% and 20% of the dry weight compared with 50% in the wild type.	[24]
*rug4*	Sucrose synthase (*SuSy1*)	Catalyzes the reversible conversion of sucrose and UDP to UDP-Glc and fructose.	Lacks detectable SuSy1 protein in the embryo. Leaves retain at least half of the normal SuSy activity. Seed mass is reduced with starch content reduced by 30%.	[25,26]
*rug5*	Starch synthase II (*SSII*)	Further elongates the intermediate glucan chains produced by SSI and is the major soluble isoform of starch synthase in pea embryos.	Abnormal starch granule morphology, and amylopectin structure with fewer chains of intermediate length and more very short and very long chains.	[27]

ADP-Glc, Adenosine 5′-diphosphate-glucose; DP, Degree of polymerization; Glc-1-P, Glucose-1-phosphate; Glc-6-P, glucose-6-phosphate; UDP-Glc, UDP-glucose.

## Abbreviations

ADP-Glc	Adenosine 5′-diphosphate-glucose.
AGPase	ADP-Glc pyrophosphorylase
CBM	Carbohydrate binding module
CLD	Chain length distribution
DBE	Starch debranching enzyme
DP	Degree of polymerization
DSC	Differential scanning calorimetry
GBSS	Granule bound starch synthase
Glc	Glucose
Glc-1-P	Glucose-1-phosphate
Glc-6-P	Glucose-6-phosphate
GWAS	Genome wide association study
HXK	Hexokinase
INV	Invertase
ISA	Isoamylase
MOS	Malto-oligosaccharide
3-PGA	3-phosphoglyceric acid
PGM	Phosphoglucomutase
PUL	Pullulanase
QTL	Quantitative trait locus
SBE	Starch branching enzyme
SNPs	Single nucleotide polymorphisms
SP	Starch phosphorylase
SPS	Sucrose phosphate synthase
SS	Starch synthase
SuSy	Sucrose synthase
TF	Transcription factor
UDP-Glc	Uridine diphosphate glucose
UGPase	UDP-glucose-pyrophosphorylase
